# Barriers to accessing radiation therapy in Canada: a systematic review

**DOI:** 10.1186/1748-717X-7-167

**Published:** 2012-10-12

**Authors:** Caitlin Gillan, Kaleigh Briggs, Alejandro Goytisolo Pazos, Melanie Maurus, Nicole Harnett, Pamela Catton, David Wiljer

**Affiliations:** 1 Radiation Medicine Program, Princess Margaret Hospital, Toronto, Canada; 2 Department of Radiation Oncology, University of Toronto, Toronto, Canada; 3 ELLICSR: Health, Wellness, and Cancer Survivorship Centre, Toronto, Canada

**Keywords:** Barriers, Access to treatment, Radiation therapy, Utilization, Wait times, Referral

## Abstract

**Introduction:**

Radiation therapy (RT) is effective treatment for curing and palliating cancer, yet concern exists that not all Canadians for whom RT is indicated receive it. Many factors may contribute to suboptimal use of RT. A review of recent Canadian literature was undertaken to identify such barriers.

**Methods:**

MEDLINE, CINAHL, and EMBase databases were used to search keywords relating to barriers to accessing or utilizing RT in Canada. Collected abstracts were reviewed independently. Barriers identified in relevant articles were categorized as relating to the health systems, patient socio-demographic, patient factors, or provider factors contexts and thematic analysis performed for each context.

**Results:**

535 unique abstracts were collected. 75 met inclusion criteria. 46 (61.3%) addressed multiple themes. The most cited barriers to accessing RT when indicated were patient age (n = 26, 34.7%), distance to treatment centre (n = 23, 30.7%), wait times (n = 22, 29.3%), and lack of physician understanding about the use of RT (n = 16, 21.6%).

**Conclusions:**

Barriers to RT are reported in many areas. The role of provider factors and the lack of attention to patient fears and mistrust as potential barriers were unexpected findings demanding further attention. Solutions should be sought to overcome identified barriers facilitating more effective cancer care for Canadians.

## Introduction

Radiation therapy (RT) is a highly effective treatment modality to cure and palliate cancer. As noted by the Chair of the Canadian Association of Radiation Oncology’s Manpower Committee, 100,000 courses of RT were administered in Canada in 2010, at more than 35 radiation treatment facilities, and yet there is concern that not all Canadians for whom RT might be indicated are receiving it.

In a 2009 Cancer System Quality Index (CSQI) report, Ontario adopted a benchmark for RT utilization, based on the estimate that 48% of those receiving a cancer diagnosis would require RT at some point in the course of their disease [1,2]. Studies in other countries have made similar estimates [3,4]. Current Canadian utilization rates fall below this benchmark, as can be suggested by the fact that 32.8% of those diagnosed with cancer in Ontario, and 31.0% nationally, are receiving a course of RT within two years of diagnosis [5]. A number of factors may be contributing to suboptimal RT utilization rates, from those attributable to the health care system [6-8] to those relating directly to the patient and the provider [9-14].

In order to better understand barriers to access and use of this important cancer treatment modality, a review of the recent Canadian literature was undertaken.

## Methods

### Definitions

Guiding definitions of both “barrier” and “access” were established. The definition of access was modified from Turnock [15], and was equated with a consultation for RT. To ensure a comprehensive assessment of factors affecting access, the definition of “barrier” was broadly determined to be anything potentially impeding access.

### Search of the literature

The MEDLINE, CINAHL, and EMBase databases were used to search keywords relating to barriers to accessing or utilizing RT in Canada. A list of keywords was created by investigators in collaboration with a medical librarian, and was informed by a previous preliminary search conducted by investigators. The initial search was conducted within the timeframe of 1980 through July 2011.

Efforts were made to limit the search to articles reporting on work conducted in the Canadian context to ensure that the results were relevant to the Canadian healthcare system and population.

### Initial categorization

Citations were collected using EndNote, and abstracts were categorized preliminarily, according to predetermined inclusion and exclusion criteria (Table 1). Four independent reviewers categorized all abstracts, and inclusion was determined based on consensus from at least two of four reviewers.

**Table 1:** Inclusion and exclusion criteria

***Inclusion criteria***	***Exclusion criteria***
· Conducted within the Canadian context	· Focuses only on clinical practice guidelines within radiation therapy (ie fractionation schedules)
· Discusses radiation therapy specifically	· Discusses cancer care more broadly, without specific reference to radiation therapy

### Secondary categorization

Full-text articles were sought for all citations that initially met inclusion criteria. Four reviewers categorized each article independently according to four broad categories, initially described by Morris [16]; 1) barriers relevant to the Health System Context, 2) barriers relevant to the Patient Sociodemographic Context, 3) Patient Factors, and 4) Provider Factors. Between two and four themes per category were defined based on trends identified in a preliminary literature review, and reviewers subcategorized each article. Consensus by at least two reviewers determined assignment under a given theme.

### Thematic analysis

Articles categorized under each theme were grouped together and reviewed for relevant data, common research findings, and salient insight.

## Results

### Initial categorization

**Figure 1:**
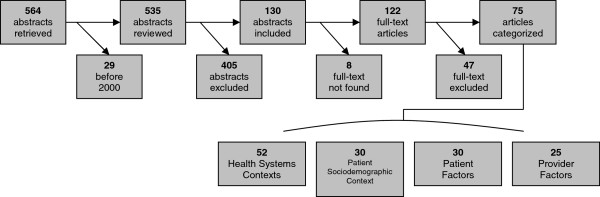
Categorization of abstracts & articles.

A flowchart of the categorization of articles is provided as Figure 1. A total of 535 unique abstracts were collected. Based on numbers and perceived relevance, a decision was made to exclude the 29 articles published before 2000, with the assumption that any relevant insight published earlier would be captured through citation in more recent studies. A further 405 abstracts were discarded upon initial review as not meeting criteria. Of those discarded, many related to psychosocial concerns of patients already receiving radiation therapy, practice patterns concerning dose and fractionation schedules, and cost analyses of RT provision. A total of 130 abstracts were thus included in the secondary thematic categorization.

### Secondary categorization

Full-text articles were found for all but 8 (6.1%) abstracts, either online or in hard-copy. No further means were undertaken to obtain the full-text of outstanding articles. Upon review of full-text, a further 47 articles were discarded as being irrelevant, most commonly as not being Canadian or referring to comparisons of radiation treatment techniques and fractionation schedules. This left 75 articles for analysis.

These articles were subsequently categorized by theme (Table 2). Forty-six of these (61.3%) addressed more than one theme, and thus a number of articles were categorized under multiple headings. In some articles, the theme was not the overall focus of the investigation and might only have been discussed briefly, but was still felt relevant by investigators.

**Table 2:** Categorization and themes of articles (n = 75)

***Category***	***n (%)***	***Barrier theme***	***n (%)***	***Reference***
Health System Context	52 (69.3%)	Distance to Treatment Centre	23 (30.7%)	[10,17-38]
		Wait Times	22 (29.3%)	[6-8,17,18,23,28,39-53]
		Treatment Centre Characteristics	17 (22.7%)	[18,33,39,41,43,46,47,54-63]
Patient Sociodemographic Context	30 (40.0%)	Race/Ethnicity	1 (1.3%)	[9]
		Socioeconomic Status	8 (10.7%)	[20,23,27,30,33,37,62,64]
		Other	21 (28.0%)	[6,10,25,28,31,36,39,41,43,46,51,61,65-72]
Patient Factors	30 (40.0%)	Age	26 (34.7%)	[6,8,11,12,20,27-33,35,38,50,51,66,71-78]
		Cultural Beliefs	1 (1.3%)	[9]
		Beliefs re: Efficacy/ Burden of Treatment	1 (1.3%)	[71]
		Life Expectancy	2 (2.6%)	[28,30]
		Other	1 (1.3%)	[25]
Provider Factors	25 (33.3%)	Lack of Referral	12 (16.0%)	[10,13,14,24,30,37,50,77-81]
		Lack of Understanding/Awareness	16 (21.3%)	[13,14,17,19,34,39,46,50,60,64,67,68,73,75,82,83]

The most identified theme was age (n = 26, 34.7%), under Patient Factors, followed by distance to a treatment centre (n = 23, 30.7%), the characteristics of the treatment centre (n = 17, 22.7%), and wait times for RT (n = 22, 29.3%), which were all barriers in the Health System Context. Provider Factors were also identified, namely a lack of understanding or awareness (n = 16, 21.6%) and a lack of referral (n = 12, 16.0%). Under the heading of Patient Sociodemographic Context, socioeconomic status was discussed in 8 articles (10.7%).

### Thematic analysis

#### Health system context

Wait times were the most frequently identified theme in the health system context. Studies investigated the length of wait times [8,23,40,43,44,49,53] and whether the length was actually a barrier to utilization [17,39,50]. The most recent wait time data found worsening wait times in Nova Scotia for breast cancer patients in 2004 [49], improved wait times in BC between 2004 and 2008 [53], and unchanged wait times between 1995 and 2005 at an institution in Toronto [44]. Looking at head and neck cancer patients in the Maritimes, Belyea et al. [40] found that between 74% and 94% of patients were waiting longer for RT between 2007 and 2009 than target standards set by Cancer Care Ontario. Despite the variability in wait times between jurisdictions and over time, subjective interviews and survey data from 3 studies found wait times to be nonetheless be identified as a barrier to RT utilization by either patients or providers [17,39,50]. Patients were potentially refusing the modality because of the wait, and providers were often not making referrals due to a perception that treatment would be delayed.

The impact of distance to treatment centres was inconsistent. Three articles support distance as a barrier [20,33,37], but only in certain scenarios. Danielson et al. [20] found it to be a barrier only in older and lower income patients and when coming from a larger community. Johnston et al. [37] found that distance was no longer a barrier once the patient had entered the RT system. The influence of distance on utilization rates was confounded by lower referral rates in remote locations, such as the BC interior and rural Ontario [10,24,50]. No studies explicitly addressed whether lower referral rates reflected patients’ reluctance to travel for RT or the physicians’ lack of consideration of RT as a treatment option.

Characteristics of RT treatment centres, including their geographical distribution, were important in considering distance as a barrier to RT [21]. Rural counties and those without facilities had lower rates for palliative RT in Ontario [33,84] and Nova Scotia [30], and for breast and lung cancers in BC [36]. Conversely, this barrier was not seen in prostate cancer in BC [36]. If the initial diagnosis was made in an academic hospital or one with an affiliated cancer centre, or if a patient’s referring physician resided in a county with a cancer centre, the patient had a significantly greater likelihood of receiving RT [19,27,33]. Tyldesley et al. [36] argued that rural areas have fewer specialists with adequate knowledge of the indications for RT, and Gray et al. [25] highlighted a lack of patient support modalities. Notably, patient outcomes were not different between the high-volume and low-volume institutions [45].

#### Patient socio-demographic context & patient factors

Themes elucidated in these categories were often interrelated, and tended to cluster around age and socioeconomic status. Referral rates were inversely related to age. While the magnitude of difference varied, older patients were found to be less likely to be referred for RT in a variety of contexts, jurisdictions, and disease sites (Table 3). In most instances, multivariate analyses demonstrated increasing age and comorbidities to be independent predictors of non-referral, and even non-treatment with respect to RT. As noted by Tyldesley et al. [78], “relative decline in the use of radiotherapy for palliative and adjuvant indications is more rapid than the decline in functional status with age in the general population”.

**Table 3:** Sites & provinces where older age related to lower referral to RT

***Site***	***Province***
Breast	Alberta [8,20]
	British Columbia [66,75]
	Ontario [76]
Colorectal cancer	Ontario [77]
Endometrial	British Columbia [12]
Nasopharynx	Ontario [78]
Palliative	Nova Scotia [30,37]
	Ontario [27,33,78]
Prostate	Ontario [51]
Small-cell lung	British Columbia [11]

The relationship between income and access to treatment was discussed in eight articles, but the relationship was unclear. Those from lower income areas generally had lower rates of consultation and utilization [20,30,33,37,62]. However, this relationship was found to be statistically insignificant on univariate analysis in another study [27]. In yet another, it was noted that income extremes had a much greater effect on rural populations in California than on similar populations in Ontario [69]. In some of these studies, there was a confounding variable of distance from treatment centre (often associated with lower income) that was not always controlled for in the analyses. Interestingly, lower income conferred a slight benefit in Quebec, where there was found to be an association with shorter wait times [23].

Other patient-dependent barriers included education level, associated with longer RT wait times [23], and life expectancy, associated with lower rates of consultation and treatment for palliation [30,37]. Patient refusal was also noted as a barrier [9,71], though it was ill-defined in the study by Puts et al. [71], as patients were not asked for their reasons for refusing treatment. The authors did note that it was most often older patients living alone, potentially refusing for such reasons as a lack of physician recommendation or not perceiving a need. Chinese women in BC refused adjuvant RT after breast-conserving surgery more than South Asian or Iranian women. This was attributed to the desire expressed by Chinese women “to be rid of their cancer” in a way they did not believe possible with RT [9]. No such differences were found between ethnic groups for surgery, chemotherapy, or hormone therapy utilization. This single study suggests that cultural beliefs, ethnicity, and poor understanding of the nature of RT could be barriers to RT, though these factors were not identified elsewhere in the Canadian literature.

#### Provider factors

The major themes identified under Provider Factors were lack of referral and poor knowledge or awareness about RT. These themes are integrally related and were often the underlying issue identified in the discussion of other barriers. Higher referral rates were associated with having a university academic appointment, being a specialist in a cancer centre, performing a higher volume of surgeries for cancers potentially requiring adjuvant RT, being more knowledgeable about RT, or having formal training in RT [13,14,19,30,82]. It was particularly noteworthy to find that physicians lacking certification by the Royal College of Physicians and Surgeons of Canada had higher referral rates for neoadjuvant RT for rectal cancers, being more likely to refer all patients rather than only those with certain clinical indications [19]. In a retrospective chart review in Nova Scotia, Lavergne et al. [30] found the difference between referral and utilization rates varied greatly between certain disease sites. The difference was high in Head and Neck cancers, where there were often concerns about comorbidities, and those referred were often not deemed eligible for treatment. For those who had received previous RT, referrals almost always led to RT utilization. A study in British Columbia noted that “the referral system acts as the initial gateway to the cancer system. The [British Columbia Cancer Agency] can only provide appropriate care to those who are referred” [24].

Low referral rates can be attributed in part to limited RT-related knowledge of the referring physician. Self-reported knowledge was poor in the majority of survey respondents in three separate investigations [13,34,82]. Poor self-rated knowledge was also correlated with poor actual knowledge in terms of indications for RT, quality of life considerations, and potential treatment effectiveness. A study in Nova Scotia found that patients referred for palliative RT tended not to be referred a second time, suggesting that providers were under a mistaken belief that RT could only be used once [30]. Another issue for referring physicians was not understanding the RT referral process, or not knowing who to contact to make a referral [13,34,85]. The understanding of patient preference relating to RT was also considered. Tucker et al. [14] reported that 51% of Canadian paediatric oncologists believed palliative RT was underutilized for reasons such as family reluctance, distance, doubt about effect on quality of life, and concerns regarding toxicity, but the authors expressed concerns that some of these beliefs might be misinformed. Tyldesley et al. [78] confirmed that “physicians can be inaccurate in the judgment of patient preference” (p479).

Only 16% of family physicians in Eastern Ontario [13] and 62% of Canadian paediatric oncologists [14] reported having had any formal RT training. The scope of this education ranged from a single lecture to a year-long clinical rotation for the family physicians, and lasted a median of 4 weeks in duration for the paediatric oncologists. Samant et al. [13] reported “lack of education [amongst family physicians in Eastern Ontario]…. to be a major barrier to radiotherapy” (p662). Indeed, 94% of those with training made referrals, as opposed to 73% of those without [14]. The majority of providers in various contexts agreed that they might benefit from more information about RT [13,34], with one study noting that there would be a preference for small group sessions to accomplish this [34]. Samant et al. [13] made the important point that educating family physicians might have a high impact for few resources.

## Discussion

In this review of Canadian literature, no single study attempted to comprehensively identify or quantify all barriers to access to treatment. The existence of similar but differently-focused reviews and the variety of issues identified suggest the potential value of completing such a study in future. While such factors as patient age, distance to treatment centre, and wait times were prevalent and expected, the role of the provider as a barrier to RT, and the lack of information pertaining to patient fears and misbeliefs are of particular interest, as they were unexpected findings in this review.

Patients must be referred in order to receive RT, and low referral rates were frequently noted in the Canadian literature, though rarely as the primary focus of an article. A lack of referral for RT was an underlying issue in the discussion of a number of health systems factors, such as distance to treatment and wait times. It also appears that the referring physician may decide not to refer due to beliefs that the patient would refuse or be inconvenienced. A related issue was a lack of understanding of RT. The few studies that attempted to rate the knowledge of referring physicians about RT found that there was a general lack of formal training for those who were in a position to refer for RT. This review demonstrated that provider factors were not only barriers unto themselves, but were also contributed to a number of other health systems and patient factors barriers.

Patient-related barriers included age, income, and education level, and yet it is perhaps the least addressed barrier, patient refusal, that is the most intriguing. Patient fears and mistrust were prevalent in other jurisdictions, but were discussed only tangentially in the Canadian literature on RT. African American populations in the United States were found to be mistrustful of the field of radiation medicine [86], and populations in Vietnam and Pakistan held religious beliefs that led to refusal of RT [87,88]. It would be important to discern whether such fears and beliefs do exist amongst Canadians. In a theme acknowledged in only a single Canadian study in this review [9], but more frequently elsewhere [89,90], women of one ethnic group were found to often refuse adjuvant RT for breast cancer due to fears of efficacy and side effects. The lack of further insight into Canadians’ perceptions of radiation may reflect an important gap in the literature deserving further study.

In some instances, the barriers identified in this review were presented in the context of a proposed solution. This tended to be on an institutional basis, and most commonly concerned a dedicated palliative clinic, with an accelerated diagnostic and planning process and a centralized referral system [18,38,56,57]. Single solutions, devised to overcome multiple identified barriers, were proven to be effective initiatives. In other areas, such as the lack of formal training for providers, potential solutions were suggested but not implemented within the scope of the article. In the majority of literature reviewed, barriers were identified but authors stopped short of proposing solutions. Future work should focus on prioritizing barriers, developing implementable solutions, and performing cost/effort analyses to determine where the greatest benefit might be achieved. Education, both of patients and providers, is expected to play a significant role in devising solutions.

There are a number of limitations to this work. While the search strategy was repeatedly broadened and refined to ensure comprehensiveness, it is possible that some relevant articles were not collected here. Limiting the scope of the review to recent Canadian literature might also have led to the exclusion of important work, though these restrictions were put in place to ensure applicability to the contemporary Canadian health care context. Another potential limitation is that no statement was made as to how prevalent an issue needed to be in a given article to warrant inclusion as a barrier. Some barriers were the primary focus of some studies but only mentioned in passing in others. Given the lack of a strong cutoff point, articles were included if it was felt that a barrier was given enough attention to make a statement about its importance. Despite these limitations, it is felt that the general scope of barriers to access was identified, as well as the most salient points about trends in each.

## Conclusion

This review of the Canadian literature identified a number of barriers to access to RT in four distinct areas: health systems context, patient socio-demographic context, patient factors, and provider factors. The most often cited reasons for not receiving RT when indicated were patient age distance to treatment centre, wait times, and a lack of understanding about RT on the part of the referring physician. The fact that provider factors were often significant barriers and that patient fears and mistrust were not as prevalent as expected were themes that emerged in this review that demand further attention. If solutions can be devised that would allow patients to overcome barriers to accessing RT, utilization of this treatment modality would likely increase, thus meeting established benchmarks and ensuring more effective use of RT for cancer care for Canadians.

## Competing interests

The authors declare that they have no competing interests.

## Authors’ contributions

CG and KB developed the search strategy, with input from NH, PC, and DW. CG, KB, MM, and AP did the independent categorization of articles and thematic analysis. Manuscript was written by CG with contributions and editing from other authors. All authors read and approved the final manuscript.
